# Computational screening of chalcones acting against topoisomerase IIα and their cytotoxicity towards cancer cell lines

**DOI:** 10.1080/14756366.2018.1507029

**Published:** 2018-11-04

**Authors:** Kanyani Sangpheak, Monika Mueller, Nitchakan Darai, Peter Wolschann, Chonticha Suwattanasophon, Ritbey Ruga, Warinthon Chavasiri, Supaporn Seetaha, Kiattawee Choowongkomon, Nawee Kungwan, Chompoonut Rungnim, Thanyada Rungrotmongkol

**Affiliations:** a Faculty of Science, Program in Biotechnology, Chulalongkorn University, Bangkok, Thailand;; b Department of Pharmaceutical Technology and Biopharmaceutics, University of Vienna, Vienna, Austria;; c Institute of Theoretical Chemistry, University of Vienna, Vienna, Austria;; d Faculty of Science, Center of Excellence in Natural Products Chemistry, Department of Chemistry, Chulalongkorn University, Bangkok, Thailand;; e Faculty of Science, Department of Biochemistry, Kasetsart University, Bangkok, Thailand;; f Faculty of Science, Department of Chemistry, Chiang Mai University, ChiangMai, Thailand;; g Center of Excellence in Materials Science and Technology, Chiang Mai University, ChiangMai, Thailand;; h Nanoscale Simulation Laboratory, National Nanotechnology Center, National Science and Technology Development Agency, Pathum Thani, Thailand;; i Faculty of Science, Biocatalyst and Environmental Biotechnology Research Unit, Department of Biochemistry, Chulalongkorn University, Bangkok, Thailand;; j Faculty of Science, Ph.D. Program in Bioinformatics and Computational Biology, Chulalongkorn University, Bangkok, Thailand

**Keywords:** Chalcone, human topoisomerase IIα, ATPase assay, molecular docking, molecular dynamics simulation

## Abstract

Targeted cancer therapy has become one of the high potential cancer treatments. Human topoisomerase II (hTopoII), which catalyzes the cleavage and rejoining of double-stranded DNA, is an important molecular target for the development of novel cancer therapeutics. In order to diversify the pharmacological activity of chalcones and to extend the scaffold of topoisomerase inhibitors, a series of chalcones was screened against hTopoIIα by computational techniques, and subsequently tested for their *in vitro* cytotoxicity. From the experimental IC_50_ values, chalcone **3d** showed a high cytotoxicity with IC_50_ values of 10.8, 3.2 and 21.1 µM against the HT-1376, HeLa and MCF-7 cancer-derived cell lines, respectively, and also exhibited an inhibitory activity against hTopoIIα-ATPase that was better than the known inhibitor, salvicine. The observed ligand–protein interactions from a molecular dynamics study affirmed that **3d** strongly interacts with the ATP-binding pocket residues. Altogether, the newly synthesised chalcone **3d** has a high potential to serve as a lead compound for topoisomerase inhibitors.

## 1. Introduction

Nowadays, cancer is one of the most serious groups of diseases in the world, with the number of deaths attributed to cancers being about 8.8 million in 2015 (World Health Organization, 2017)^1^. Chemotherapy is currently a highly effective cancer treatment. However, most chemotherapeutic agents give severe side effects with limited selectivity against various cancer cells. Hence, development of anti-cancer drugs with no or less side effects and a high selectivity is of prime concern^2^. Targeted cancer therapy, in which the drugs are used to specifically block the growth of cancer by interfering with molecular targets and consequently causing less damage to normal cells, has become one of the high potential cancer treatments. Several kinds of molecular targets have been focused on in recent years, including human topoisomerase II (hTopoII). This enzyme catalyzes the cleavage and rejoining of double-stranded DNA and so it is essential in several vital cell processes, such as replication, transcription, chromosome separation and segregation^3^. Generally, hTopoII exists in two homologous structures but in different isoforms, hTopoIIα and hTopoIIβ. The hTopoIIα isoform shows a low expression level in the G cell cycle phase but an increased concentration in the S and G_2_/M phases compared to normal cells, whilst hTopoIIβ does not change its concentration during the cell cycle^4^. Since hTopoIIα is highly overexpressed in proliferating cancer cells^5^, it has gained attention from many researchers who are developing new anti-cancer drugs.

**Figure 1.**
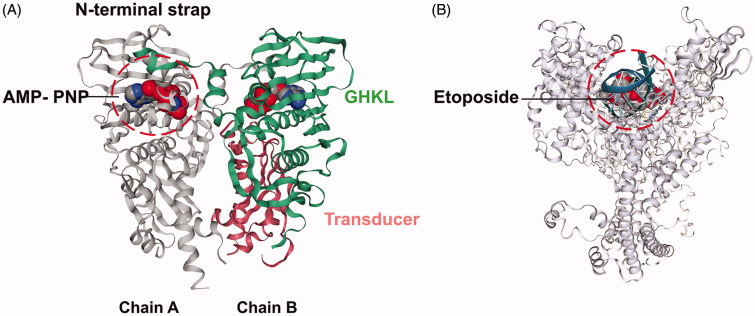
The hTopoIIα structures used in the docking study. (A) The ATPase domain of hTopoIIα with the 5′-adenylyl-β,γ-imidodiphosphate, AMP-PNP (space filling model), in the ATP-binding pocket, where the GHKL and transducer domains are shown in green and pink (PDB code: 1ZXM). (B) The hTopoIIα/DNA/etoposide ternary complex (PDB code: 3QX3).

There are two important motifs for drugs targeting hTopoIIα, namely the ATPase domain (Figure 1(A)) and the DNA-binding core (Figure 1(B))^6^. The hTopoIIα inhibitors can be divided into two categories, hTopoIIα poisons and hTopoIIα catalytic inhibitors^3^. For hTopoIIα poisons (etoposide, doxorubicin, anthracyclines and mitoxantrone), they are clinically active agents that generate a high level of hTopoII–DNA covalent complexes by stimulating cleavage of the G-segment and blocking relegation of DNA^7^. On the other hand, hTopoIIα catalytic agents (ICRF-187, novobiocin, merbarone and salvicine) affect the catalytic cycle of hTopoIIα by elimination of the enzymatic activity^8–10^. Although these different catalytic agents share the same effect, they interact with hTopoIIα at different binding sites. For example, the ICRF-187 binding pocket is located in the middle of the primary dimer interface^8^, while merbarone acts by blocking the DNA cleavage reaction of hTopoIIα. The merbarone-binding site possesses an interaction domain overlapping with that of etoposide^11–13^. Salvicine, a derivative of diterpenoid quinones isolated from the traditional Chinese medicinal plant *Salvia prionitis*^10^^,^^14^, targets the ATPase domain^15–17^.

The polyphenolic compounds, chalcones or 1,3-diphenyl-2-propene-1-ones, are precursors for flavonoids and isoflavonoids. They consist of two aromatic rings connected by an α,β-unsaturated carbon atom chain. Chalcones are naturally found in several plants, such as *Piper methysticum*^18^ and members of the *Glycyrrhiza*^19^ and *Angelica*^20^ genera. Natural and synthetic derivatives of chalcones have been reported to exert several biological activities, including anti-fungal, anti-microbial, anti-protozoal, anti-viral, anti-malarial, anti-inflammatory and antioxidant effects^21–24^. In addition, they have been shown to have cytotoxic activities against various cancer cell lines, including breast (MCF7)^25–27^, ovary (A2780)^28^, lung (A549)^27^^,^^29^, colon (SW480)^30^, liver (HepG2)^31^^,^^32^ and cervical (HeLa)^33^ cancer-derived cell lines.

**Figure 2.**
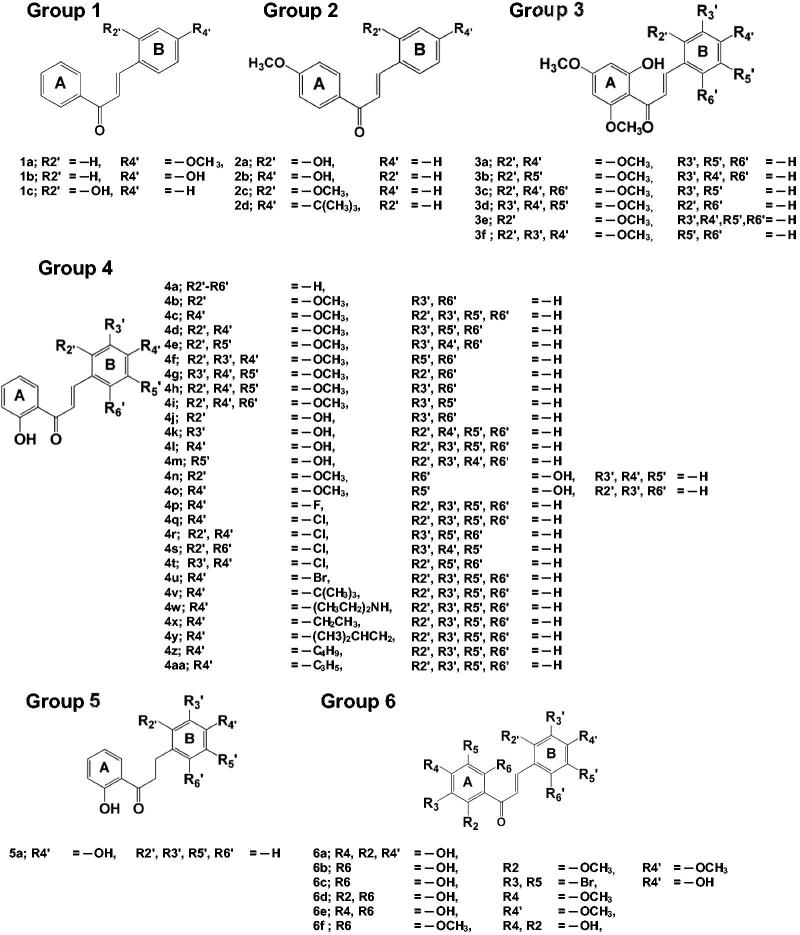
Chemical structure of the 47 designed chalcones from six different groups.

Chalcones have attracted attention because of their promising therapeutic effects, since they are able to target multiple cellular molecules, such as MDM2/p53, tubulin, proteasome, NF-κB, TRIAL/death receptors and mitochondria-mediated apoptotic pathways, cell cycle, STAT3, AP-1, NRF2, AR, ER, PPAR-γ, β-catenin/Wnt^34^ and especially hTopoIIα^24^^,^^35–37^. Moreover, epipodophyllotoxin–chalcone hybrids exhibited an enhanced *in vitro* cytotoxicity and higher topoisomerase II inhibitory efficiency than etopoiside^38^. A series of chalcone-triazole derivatives presented a promising anticancer activity against the A-549 cell line and showed high binding affinities towards DNA topoisomerase IIα and α-glucosidase targets^39^. Moreover, the novel *bis*-fluoroquinolone chalcone-like derivatives were found to inhibit both hTopoIIα and tyrosine kinase^40^. Recently, a series of 2′- and 4′-aminochalcones were found to inhibit the growth of a canine malignant histiocytic cell line (DH82) and the transcription of the hTopoIIα and TP53 genes^41^. In the present study, in order to find new potential anti-cancer agents against hTopoIIα, the new 47 chalcone derivatives were designed (Figure 2) and then screened *in silico* using a molecular docking approach. The potent chalcones with a more favorable interaction energy than that of the known hTopoIIα inhibitors were then synthesised and tested for their *in vitro* cytotoxicity towards three cell lines derived from urinary bladder (HT-1376), cervical (HeLa) and breast (MCF-7) cancers. Then, all-atom molecular dynamics (MD) simulations were performed to investigate the structure and dynamics properties as well as the ligand–target interactions between the most potent chalcone and hTopoIIα.

## 2. Methodology

### 2.1. Material

Human urinary bladder, cervical and breast cancer-derived cell lines (HT-1376, HeLa and MCF-7, respectively) were obtained from the American Type Cell Culture Collection (ATCC), Manassas, VA. Dulbecco’s modified eagle’s medium (DMEM), fetal bovine serum (FBS), penicillin–streptomycin (Pen–Strep) and trypsin were purchased from Life Technologies (Carlsbad, CA). Thiazolyl blue (MTT), dimethyl sulphoxide (DMSO), sodium dodecyl sulphate (SDS) and phosphate-buffered saline (PBS) were purchased from Sigma-Aldrich (Darmstadt, Germany). Salvicine was purchased from *Chemfaces* (Wuhan, P.R. China). The purity of the compound was more than 98.0%. All other chemicals and solvents used were of analytical grade. Plasmid pET28b-hTopoIIα-ATPase was gifted from Dr. Nonlawat Boonyalai. ADP-Glo^TM^ Kinase Assay kit was purchased from Promega (Madison, WI). All solvents used for the synthesis were purified prior to use by standard methodologies. The reagents used for synthesis were purchased from Sigma-Aldrich, Merck or TCI chemical companies and were used without further purification.

### 2.2. Computational methods

#### 2.2.1. Molecular docking

Due to the possibility of the inhibition of two motifs of the hTopoIIα (ATP-binding site in the ATPase domain and the etoposide-binding pocket in the hTopoIIα/DNA complex**)**, the predicting mode of the inhibitory activity of chalcones on both sites was studied by molecular docking using the CDOCKER module of Accelrys Discovery Studio 3.0 (Accelrys Inc, San Diego, CA, USA) as previously reported^42^. The starting structures of the 47 designed chalcone derivatives were built by the GaussView program^43^, while those of salvicine and etoposide were taken from the ZINC database^44^. To validate the docking method, the co-crystallised ligands were initially docked into the binding pocket with 100 independent runs, i.e. docking of AMP-PNP into the ATP-binding site of the hTopoIIα ATPase domain (1ZXM.pdb), and etoposide into its binding pocket of the hTopoIIα/DNA complex (3QX3.pdb). The position of docked ligands did not differ significantly from the crystallised conformation ligands (RMSD = 0.80 Å for AMP-PNP and 0.44 Å for etoposide) and so the 47 chalcones were then separately docked into both sites, while salvicine (used as the reference compound at the ATPase domain) was only docked into the ATP-binding site. The chalcones with predicted interaction energies towards hTopoIIα that were more favorable than those of the known inhibitors were synthesised and their *in vitro* cytotoxicity against the three cancer cell lines was tested (see Section 2.3.3).

#### 2.2.2. MD simulation

All-atom MD simulations under a periodic boundary condition were performed on the most potent chalcone selected from the *in vitro* cytotoxicity study (Section 2.3.3) in complex with hTopoIIα in aqueous solution, following the previously reported MD study on the binding of mansonone G to hTopoIIα^42^. The partial charges of the ligand were prepared according to standard procedures^45–47^. The ligand was optimised with *ab initio* calculation using the HF/6-31G* method in the Gaussian09 program^48^. The electrostatic potential (ESP) charges of the ligand were calculated using the same level of theory, and then the restrained ESP (RESP) charges were obtained by the charge fitting procedure using the antechamber module in the AMBER 14 package program^49^. The general AMBER force field (GAFF)^50^ and AMBER ff03 force field^51^ were applied for the ligand and protein, respectively. The protonation states of all ionizable amino acids were determined using PROPKA 3.1^52^. The complex was solvated by TIP3P water molecules^53^ within 12 Å around the system surface. Chloride ions were introduced to neutralise the total positive charge of the chalcone/hTopoIIα complex.

To remove the bad contacts and steric hindrances, the added hydrogen atoms were minimised with 1000 steps of steepest descents (SD) followed by 2000 steps of conjugated gradients (CG) using the Sander module in AMBER 14. The water molecules and ions were then minimised with 500 steps of SD followed by 500 steps of CG, while a 500 kcal/mol Å^2^ force constant was used to restrain hTopoIIα. The whole system was then fully minimised with 1000 steps of SD and CG. All covalent bonds involving hydrogen atoms were constrained by the SHAKE algorithm (Amber, San Francisco, CA)^54^. The long-range electrostatic interactions were calculated according to the Particle Mesh Ewald (PME) approach^55^ with a cutoff distance of 12 Å for non-bonded interactions.

The system was heated to 310 K for 100 ps and then simulated at the same temperature for 80 ns in the NPT ensemble using a time step of 2 fs. The MD trajectories in the production phase were taken for analysis in terms of the per-residue decomposition free energy and intermolecular hydrogen bonds (H-bonds) between the ligand and hTopoIIα using the MM/PBSA.py and cpptraj modules, respectively. The percentage of H-bond occupation was calculated using the two criteria of: (i) the distance between proton donor (HD) and acceptor (HA) atoms ≤3.5 Å and (ii) the angle of HD-H…HA >120°.

### 2.3. Experimental approach

#### 2.3.1. Synthesis of chalcone derivatives

The three selected chalcones (**3c**, **3d** and **3f**) were synthesised by Claisen–Schmidt condensation with some modifications between selected acetophenones and benzaldehydes under a basic condition, according to the procedures described by Cabrera^56^. The target products were purified by column chromatography and their structures were elucidated by NMR spectroscopy.

#### 2.3.2. Cell culture and sample preparation

Stock cultures of HT-1376, HeLa and MCF-7 cell lines were grown in T-75 flasks in complete medium [CM; DMEM, 10% (v/v) FBS and 1% (v/v) Pen–Strep] at 37 °C under 5% (v/v) CO_2_. They were subcultured once a week, for HeLa and MCF-7 cells at a 1:100 ratio and for HT-1376 at 1:20 ratio by washing with PBS and then the cells were detached with trypsin. The 10^−1^ M stock solution of each respective chalcone derivative was prepared in 100% DMSO.

#### 2.3.3. Cytotoxicity assay

The cytotoxicity of the chalcones and salvicine was measured according to a published method^57^ with some modifications. The cell viabilities of three cancer cell lines (HT-1376, HeLa and MCF-7) exposed to the screened chalcone derivatives were evaluated by the MTT assay. The cell suspension (100 μL) was seeded into 96-well plates at a density of 2 × 10^6^ cells/well and then incubated for 24 h under normal culture conditions before the addition of the respective test compound at various concentrations [100, 50, 25, 12.5 and 0 (control) μM] and incubated for another 24 h. Then, 10 µL of fresh MTT solution (5 mg/mL) was added to each well and incubated at 37 °C for 2 h, before the reaction was stopped by adding 100 μL of DMSO. The absorbance was measured at 570 nm with correction for background at 690 nm using a microplate spectrophotometer system (Infinite M200 micro-plate reader, Tecan, Männedorf, Switzerland). The amount of the colored product is assumed to be directly proportional to the number of viable cells. Each experiment was performed in triplicate and repeated three times. The percentage cell viability in each compound was calculated relative to the control, and the IC_50_ values were determined in comparison with untreated controls using the Table Curve 2D program version 5.01 (Systat, San Jose, CA).

#### 2.3.4. Expression and enrichment of the recombinant (r)hTopoIIα ATPase domain

Expression and enrichment of the rhTopoIIα ATPase domain was modified from that reported^58^. The expression plasmid pET28b-hTopollα-ATPase was transformed into *Escherichia coli* BL21 (DE3) cells and a transformant colony was selected for large-scale protein expression and grown at 37 °C to an optical density at 600 nm of ∼0.6 in LB broth (2 L) containing 50 µg/mL kanamycin. Protein expression was then induced by adding 0.5 mM IPTG at 30 °C for 5 h. The cells were harvested by centrifugation at 6000×*g*, 4 °C and resuspended in lysis buffer [50 mM Tris–Cl pH.8, 0.5 M NaCl, 5 mM imidazole, 0.5% (v/v) Triton X-100, 1 mM PMSF] and lysed by sonication. After clarification by centrifugation (as above) the supernatant was harvested, and the rhTopoIIα-ATPase enriched for using HisTrap Chelating HP and Resource S column chromatography, eluting in exchange buffer [50 mM Tris pH.7.5, 50 mM NaCl, 5% (v/v) glycerol, 50 mM KCI, 5 mM MgCl_2_] from a PD-10 desalting column. The enriched protein was analyzed by 12% sodium dodecyl sulphate–polyacrylamide gel electrophoresis (SDS–PAGE) and stained by Coomassie blue.

#### 2.3.5. ATPase assay

The inhibitory activities of salvicine and chalcone **3d** were determined by measuring the ATPase activity of rhTopoIIα-ATPase using the ADP-Glo™ Kinase Assay. Briefly, 8 µL of buffer (40 mM Tris–HCI pH 7.5, 20 mM MgCl_2_, 0.1 mg/mL BSA) was added to each well of a 384-well plate (Promega, solid white) with 5 µL of enzymes (10 ng/µL) and 2 µL of the test compound at different concentrations. The reaction was initiated by the addition of 5 µL of 12.5 µM ATP, allowed to proceed for 1 h at room temperature and then stopped by the addition of 5 µL of ADP-Glo™ Reagent and incubating at room temperature for 40 min. Next, 10 μL of Detection Reagent was added and incubated for 30 min prior to the addition of luciferase and luciferin to detect the ATP by measuring the luminescence of each well with a microplate spectrophotometer system (Synergy HTX Multi-Mode reader, BioTek, Winooski, VT). All assays were performed in triplicate. The percentage relative inhibition of salvicine and **3d** was calculated as shown in Equation (1);

(1) $$\text{\% relative inhibition }=\frac{[\left(\text{positive}-\text{negative}\right)\;-\;(\text{sample}\;-\;\text{negative}\;)]\times \;100}{\;(\text{positive}\;-\;\text{negative})},$$

where negative and positive are the luminescence without and with the enzyme activity, respectively, and sample is luminescence with the sample. Finally, the IC_50_ curve was determined by GraphPad Prism version 6 (GraphPad Sofware, La Jolla, CA).

## 3. Results and discussion

### 3.1. Molecular docking studies

**Figure 3.**
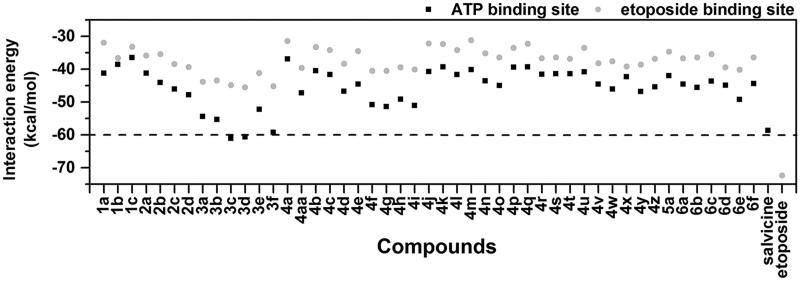
CDOCKER interaction energies (kcal/mol) of the designed chalcone derivatives binding at two different sites relative to the known hTopoIIα inhibitors, salvicine and etoposide.

To investigate the most favorable binding site of the 47 designed chalcones, each compound was separately docked into the ATP-binding site in the ATPase domain of hTopoIIα and the etoposide-binding pocket in the hTopoIIα/DNA complex. The predicted interaction energies of all chalcones at both sites were plotted and compared with those of salvicine and etoposide (Figure 3). The interaction energies of the chalcones ranged from −45.6 to −32.4 kcal/mol in the etoposide-binding pocket and from –60.0 to −37.5 kcal/mol in the ATP-binding site. This suggested that all the chalcone derivatives specifically interacted with the ATPase domain rather than with the hTopoIIα/DNA complex.

**Figure 4.**
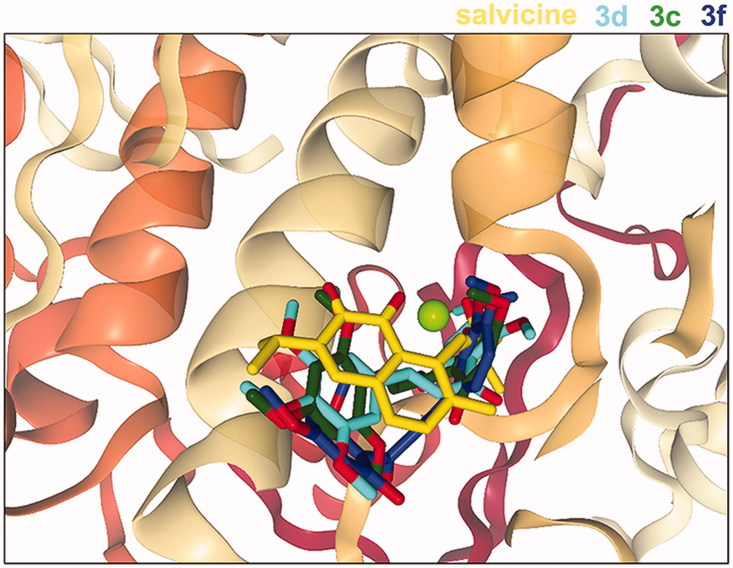
Superimposed structures of the three most active chalcones (**3c**, **3d** and **3f**) from the docking study with that of salvicine in the ATP-binding pocket of the hTopoIIα ATPase domain. Figure created by NGL viewer (http://nglviewer.org/ngl/).

Among all 47 chalcones, the group-3 compounds (**3c**, **3d** and **3f**) showed high interaction energies with the hTopoIIα ATPase domain (−61.1, −60.7 and −59.3 kcal/mol), which were better than that of salvicine (−58.7 kcal/mol) at the hTopoIIα ATPase domain. However, none of the tested chalcones were stronger than etoposide binding in the hTopoIIα/DNA complex (−72.4 kcal/mol). Additionally, the mode of action of these three compounds was likely comparable with salvicine in the ATP-binding pocket (Figure 4). The important residues that contributed to ligand stabilisation via van der Waals (vdW) and H-bond interactions are summarised in Table 1. There are at least four conserved residues between each chalcone and salvicine. The obtained results were similar to the docking study of 4-ethoxycarbonylmethyl-1-(piperidin-4-ylcarbonyl)-thiosemicarbazidehydrochloride, and napthoquinone-containing compounds, which specifically targeted the ATPase domain^42^^,^^59^. Since **3c**, **3d** and **3f** may be effective as ATP competitors at the ATP-binding site of the hTopoIIα ATPase domain, these three compounds were synthesised and their *in vitro* cytotoxicity towards three cancer cell lines was then tested.

**Table 1.** Contact residues of the hTopoIIα ATPase domain for the binding of salvicine and three chalcones (**3c**, **3d** and **3f**). The residues in bold format stabilise the ligand binding via H-bond interaction, while the conserved residues between each chalcone and salvicine are shown in underlined format.

Compound	Structure	Contact residues
Salvicine		**N91,** S148, S149, R162, G164, **A167** and K168
**3c**		N91, R98, G164, G166 A167 and ****K168****
**3d**		N91, D94, T147, S148, ****S149**,** N150, G164, **G166** and ****K168****
**3f**		N91, R98, S148, S149, N150, G164, ****A167**** and ****K168****

### 3.2. Cytotoxicity towards cancer cell lines

After screening the potent chalcones for inhibition of hTopoIIα by molecular docking, the three compounds that exhibited better interaction energies than salvicine (**3c**, **3d** and **3f**) were selected for synthesis to test their cytotoxicity on the HT-1376, HeLa and MCF-7 cancer-derived cell lines using the MTT assay. The derived IC_50_ values of the three chalcone derivatives and salvicine on the three cancer cell lines are summarised in Table 2. All three screened chalcones showed a higher cytotoxicity to all three cell lines than salvicine, with **3d** being the most cytotoxic with an IC_50_ value of 10.8 ± 1.1, 3.2 ± 2.2 and 21.1 ± 6.3 µM against the HT-1376, HeLa and MCF-7 cell lines, respectively. The IC_50_ of salvicine in a lung cancer cell line (A549) was previously reported to be 18.66 µM^60^. The diversity of the cytotoxicity of these three chalcones could suggest that the position of the methoxy group on the B ring of the chalcones affected the cytotoxicity. The methoxy groups substituted at the R_2_, R_3_ and R_4_ positions were found to be most important in terms of anti-cancer activities. Moreover, the different IC_50_ values of the chalcone derivatives in each cancer cell line may reflect the different expression levels of hTopoIIα and proliferation rates between those cell lines^61–64^. Cells containing a high concentration of hTopoIIα are more sensitive to hTopoIIα-inhibiting drugs than cells containing a lower concentration of hTopoIIα^61^^,^^65^. Thus, these chalcones might inhibit HeLa cells better than MCF-7 and HT-1376 cells because of the higher hTopoIIα levels typically expressed in cervical cancer cells than in breast and urinary bladder cancer cells^66^. Considering the data from the *in silico* molecular docking and the *in vitro* cytotoxicity against cancer cell lines, it is possible that **3d** tends to inhibit the hTopoIIα ATPase domain in a somewhat similar manner as salvicine. However, to gain additional information about the inhibition of hTopoIIα at the ATPase domain by salvicine and **3d**, their *in vitro* inhibitory activity against the ATPase activity of rhTopoIIα was evaluated.

**Table 2.** *In vitro* IC_50_ values of the three chalcone compounds and salvicine against the HT-1376, HeLa and MCF-7 cancer-derived cell lines and the rhTopoIIα ATPase domain.

Compound	IC_50_ value (µM) against			IC_50_ against rhTopoIIα ATPase domain (nM)
	HT-1376	HeLa	MCF-7	
**3c**	46.1 ± 4.2	30.9 ± 1.3	38.6 ± 1.4	N/T
**3d**	10.8 ± 1.1	3.2 ± 2.2	21.1 ± 6.3	7.5 ± 4.2
**3f**	92.0 ± 1.8	21.2 ± 8.7	72.1 ± 3.8	N/T
Salvicine	106.5 ± 4.7	70.1 ± 4.5	>200	326.5 ± 6.6

N/T: non-tested.

### 3.3. Inhibition of the hTopoIIα ATPase domain

**Figure 5.**
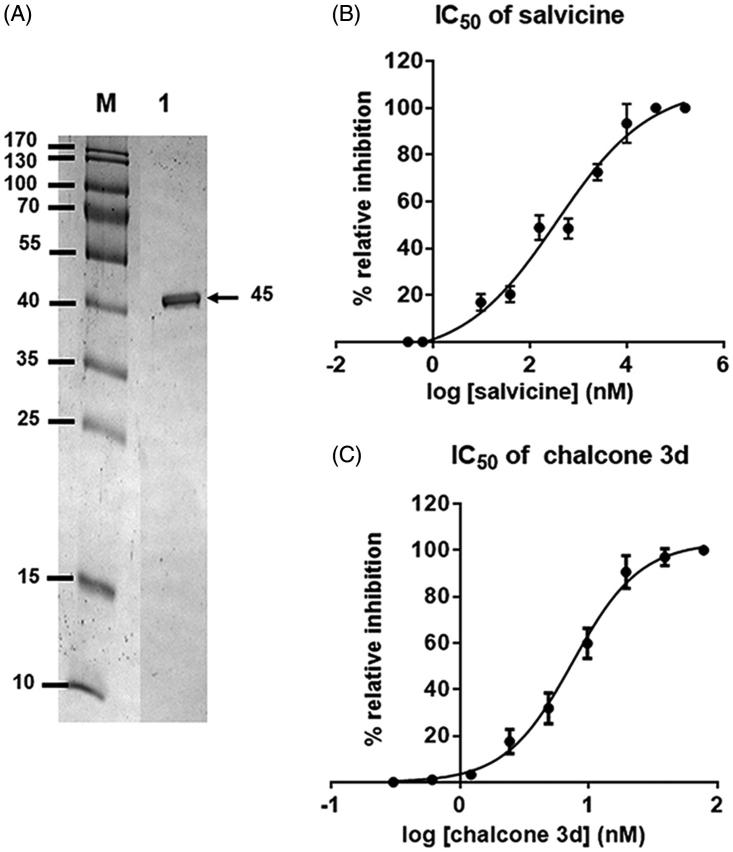
(A) SDS–PAGE gel analysis of the enriched rhTopoIIα ATPase domain. Lane M: molecular weight marker of standard protein; Lane 1: enriched rhTopoIIα ATPase domain (45 kDa). **(**B,C) The IC_50_ curves of (B) salvicine and (C) **3d** against the ATPase activity of **r**hTopoIIα. Data are shown as the mean ±1 SD, derived from three independent experiments.

In order to assess the inhibition of ATPase activity by salvicine and **3d**, the rhTopoIIα ATPase domain was expressed from the pET28b-expression vector and enriched by following a previously reported protocol^58^ for use in the ATPase enzymatic activity assay. The rhTopoIIα ATPase domain was enriched to apparent homogeneity, with the 45 kDa ATPase domain evident as a single band following SDS–PAGE resolution and Coomassie blue staining (Figure 5(A)). The ATPase inhibitory activity of different concentrations of salvicine and **3d** was then comparatively studied using a commercial kit (ADP-Glo™ Kinase Assay, see also in material). The obtained IC_50_ curves of salvicine and **3d** are shown in Figure 5(B,C), respectively, and listed in Table 2. The chalcone **3d** showed an ATPase inhibitory activity with an IC_50_ value (7.5 nM) that was some 43.5-fold lower than that for salvicine (326.5 nM). To investigate the binding and interaction of **3d** against hTopoIIα at the ATPase domain a detailed investigation of the **3d**/hTopoIIα complex in aqueous solution was then performed in silico using MD simulations.

### 3.4. MD simulations

All-atom MD simulations were performed on the docked **3d**/hTopoIIα complex with three different velocities for 80 ns to understand the structure and dynamics of **3d** at the ATP-binding site of the hTopoIIα ATPase domain. Since the **3d** binding patterns and interactions with hTopoIIα obtained from three different simulations were similar, the results presented here are taken from one representative simulation. The root mean square displacement (RMSD) plot in Supplementary Figure S^1^ showed that the **3d**/hTopoIIα complexes had reached equilibrium by 50 ns. Herein, the snapshots taken from the last 10-ns were extracted for analysis in terms of the binding pattern and ligand–protein interactions as follows.

**Figure 6.**
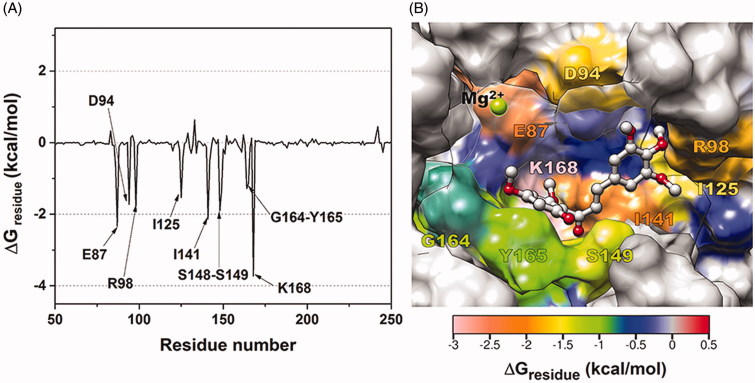
(A) Per-residue decomposition free energy of **3d/**hTopoIIα complex and (B) the binding orientation of **3d** inside the ATP-binding pocket of ATPase domain, drawn from the last MD snapshot.

In order to elucidate the hTopoIIα ATPase residues important for **3d** inhibition at the ATP-binding site, the per-residue decomposition free energy (ΔG_residue_) was evaluated by the MM/PBSA approach using the 100 snapshots over the last 10-ns simulation. The results are given in Figure 6(A), where the binding orientation of **3d** inside the ATP-binding pocket with the contour energy of residue contribution is drawn in Figure 6(B). The fingerprint in Figure 6(A) showed only residues 50–250 in chain A, while the rest of protein (chain A residues 29–49 and 251–405 plus all chain B residues) had no interaction with the ligand. The negative and positive ΔG_residue_ values represented the degrees of stabilisation and destabilisation for ligand binding, respectively.

**Figure 7.**
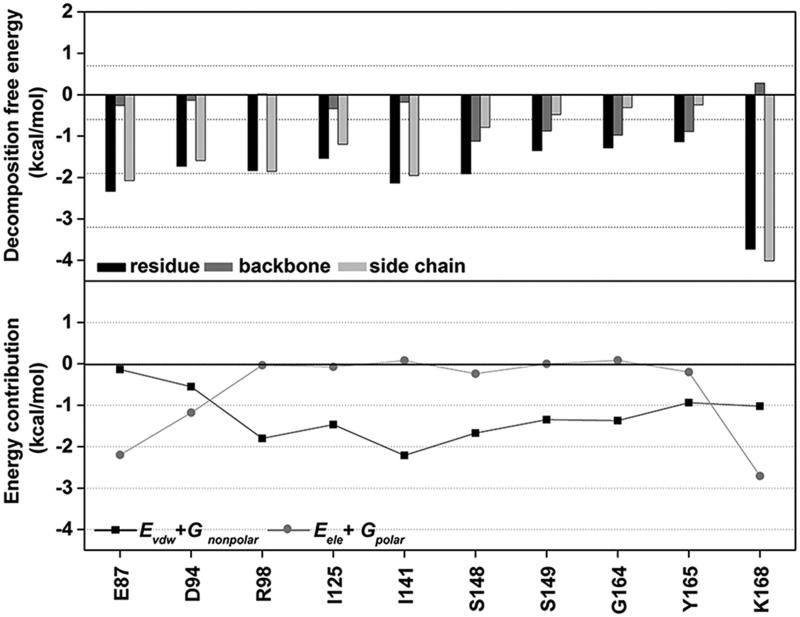
(Top) Per-residue decomposition free energy of the 10 key residues (black) and their contribution from backbone (dark gray) and side chain (light gray). (Bottom) The vdW (*E_vdW_* + *G_nonpolar_*) and electrostatic (*E_ele_* + *G_polar_*) energy contributions are given in black and gray lines, respectively.

From Figure 6(A), 10 residues preferentially stabilised **3d** with an energy contribution lower than −1.0 kcal/mol: E87, D94, R98, I125, I141, S148, S149, G164, Y165 and K168. This implies that these residues probably play a crucial role in **3d** binding to the ATPase domain. The free energy contributions of each key residue, decomposed to backbone and side chain as well as electrostatic (E_ele_+G_polar_) and vdW (E_vdW_+G_nonpolar_) terms, are plotted in Figure 7. Most of the important residues support the **3d** binding via the vdW energy contribution, while E87, D94 and K168 residues likely presented the electrostatic contribution. The strongest energy stabilisation for **3d** (−3.8 kcal/mol) came from the K168 residue. In contrast, it has been reported that the K168 was not interfere with salvicine binding and even destabilised some mansonone G compounds in the ATP-binding pocket^42^. However, the observed binding patterns of **3d** in this work are somewhat similar with salvicine (E87, I125 and I141) and mansonone G (D94, I125, I141 and G164) in our previous work.^42^

**Figure 8.**
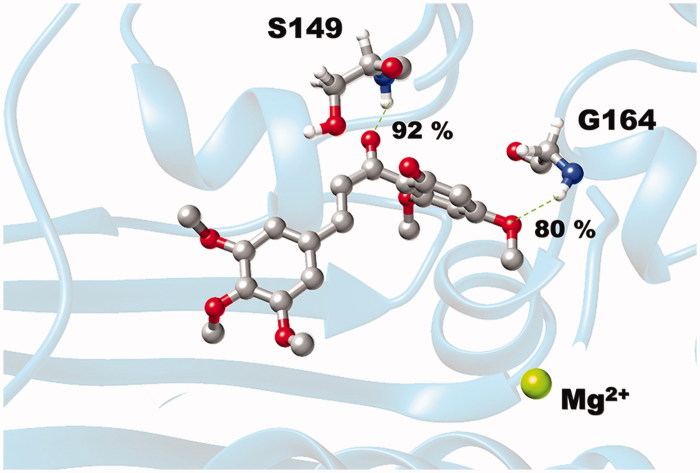
Hydrogen bond formation between chalcone **3d** and the two residues in the ATP-binding pocket of hTopoIIα ATPase domain, where the percentage of H-bond occupation is also given.

The results also demonstrated that the **3d** binding energy is mainly contributed from the side chains of the key residues (E87, D94, R98, I125, I141, S148, S149, G164, Y165 and K168), except for the S148, S149, G164 and Y165 residues where the ligand–protein interactions substantially come from their backbone contributions. The information was well supported by the formation of two strong H-bonds between the carbonyl group of **3d** and the backbone nitrogen of S149 (92%) as well as the 3-methoxy group on its A-ring and the backbone nitrogen of G164 (80%), (see intermolecular H-bonds between **3d** and hTopoIIα residues in Figure 8).

## 4. Conclusions

A series of 47 designed chalcones were screened *in silico* as potent anti-cancer agents by computational methods. Molecular docking of the chalcone derivatives relative to salvicine, a known inhibitor of hTopoII at the ATPase domain, suggested that the ATP-binding site of hTopoIIα ATPase domain serves as the target site for the considered chalcones. The three most active chalcones (**3c**, **3d** and **3f**) had interaction energies towards the ATPase domain that were stronger than that of salvicine. Compound **3d**, containing 2,4-dimethoxy and 6-hydroxy groups on A ring and 3′,4′,5′-trimethoxy on the B ring, showed the highest *in vitro* cytotoxicity against the HT-1376, HeLa and MCF-7 cancer cell lines. Moreover, **3d** inhibited the rhTopoIIα ATPase activity *in vitro* with an IC_50_ value some 43.5-fold lower than that for salvicine. From 80-ns MD simulations of the **3d**/hTopoIIα complex, the key residues responsible for **3d** binding via vdW and electrostatic interactions were E87, D94, R98, I125, I141, S148, S149, G164, Y165 and K168. The residue K168 exhibited the strongest energy stabilisation for **3d**, while residues S149 and G164 formed two strong H-bond interactions with the carbonyl and 3-methoxy groups of **3d**. In summary, the in silico and *in vitro* results suggested that **3d** can serve as a lead compound for further anti-cancer drug development.

## Acknowledgements

The authors would like to thank Robert Butcher for constructive criticism and English language improvement of the manuscript. K.S. thanks the Thailand Graduate Institute of Science and Technology [TGIST Grant No. TG550958052D], the 90th Anniversary of Chulalongkorn University Fund (Ratchadaphiseksomphot Endowment Fund), and the Overseas Presentations of Graduate Level Academic Thesis from Graduate School. N.K. thanks the Center of Excellence in Materials Science and Technology, Chiang Mai University for financial support. P.W. thanks Chulalongkorn University. Through travel grants for a short research visit, this research was also supported by the ASEAN-European Academic University Network (ASEA-UNINET). The Computational Chemistry Center of Excellent, and the Vienna Scientific Cluster (VSC-2) are acknowledged for facilities and computing resources.

## Supplementary Material

Supplementary_result.pdf
