# Supplementary material for: Computational screening of chalcones acting against topoisomerase IIα and their cytotoxicity towards cancer cell lines
Source: J Enzyme Inhib Med Chem. 2018 Nov 4;34(1):134–43. doi: 10.1080/14756366.2018.1507029 (PMC6225485; doi:10.1080/14756366.2018.1507029)
Supplement: Supplementary_result.pdf [file IENZ_A_1507029_SM0866.pdf]

Supplementary result

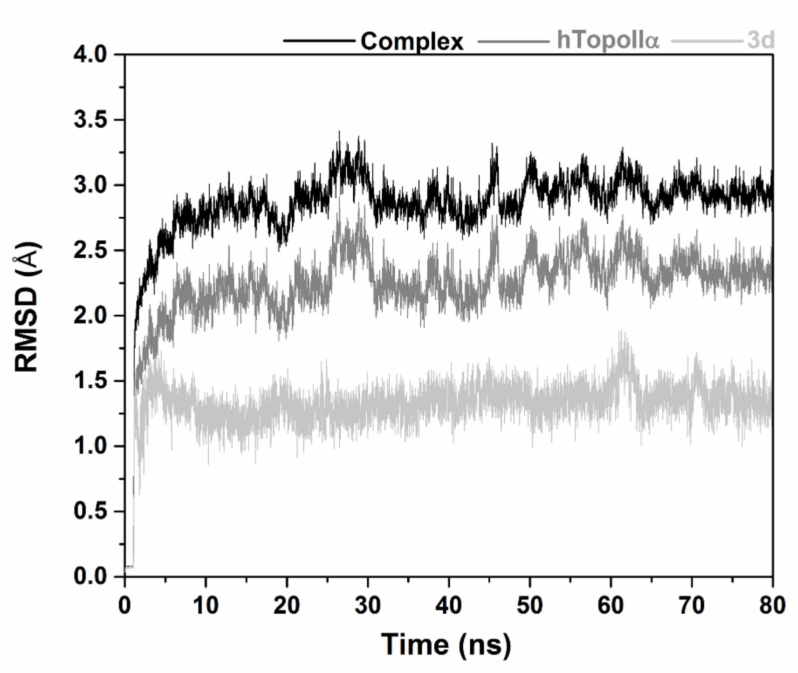

Fig. S1 RMSD plots during 80 ns MD for the simulated systems of chalcone **3d**/hTopoIIα
